# Controlling Malaria on the Road to Elimination: A Commentary from Kent Campbell Written in 2014

**DOI:** 10.4269/ajtmh.24-0628

**Published:** 2024-10-29

**Authors:** Carlos C. Campbell, Kammerle Schneider, Richard W. Steketee

**Affiliations:** ^1^ Center for Malaria Control and Elimination, Malaria Control and Elimination Partnership in Africa, PATH, Seattle, Washington;; ^2^ Independent consultant, Bethesda, Maryland

Editor’s note: Carlos C. (Kent) Campbell, a long-term leader in public health and President of the ASTMH in 2007, died in February 2024 following a long series of illnesses. In 2014, after over 40 years leading efforts toward the control and elimination of malaria, Dr. Campbell authored this perspective piece, but it was never submitted for publication. While the perspective is now 10 years old, it remains relevant to discussions and planning that continue today. Indeed, since this was written, efforts to eliminate malaria have succeeded in some regions, but sadly become stalled in others, especially in sub-Saharan Africa. Thus, the topic remains highly relevant, and the perspective is published to share with readers Dr. Campbell’s wisdom regarding what remains one of our greatest topical medicine challenges.

Over the last fifteen years malaria has captured the attention of the public health community, researchers, global and national funders, and most importantly, national governments and communities in endemic areas.^1^ Renewed efforts to fight the disease have resulted in an unprecedented 50% reduction in malaria deaths in African children since 2000. To maintain momentum and stay ahead of resistance and resurgence, many endemic countries and global financing organizations are now championing the development of country programs that are more ambitious than just trying to impact on malaria deaths and illnesses – comprehensive programs that are explicitly targeting elimination of malaria transmission.

In the midst of this renewed commitment, there has been confusion in the malaria community about the appropriate goal for these programs. It has been proposed that national programs can either opt for malaria elimination, working towards ending transmission of the parasite altogether or alternatively settle for malaria control, sustaining high levels of programmatic effort and intervention coverage indefinitely.^2^ Some assert that elimination is too expensive and too ambitious an aspiration for many countries at this point. Malaria control may be invoked as a safer, more feasible goal for national governments to embrace.^2^^–^^4^ This position, however, represents a fundamental misunderstanding of the strategies needed in the malaria fight and what it means to have a goal.

Aggressive implementation of malaria intervention approaches, like the scale-up for impact (SUFI^‡^) approach^5^ has certainly accelerated the reduction of disease burden through expanding the ownership and use of proven interventions like insecticide treated mosquito nets and quality case management. But a SUFI coverage target was not intended to be a program goal in itself; rather, it is the initial phase in accelerating and maximizing the impact of national programs.^6^

Attempting to simply maintain these relatively high-cost, and certainly high intensity programmatic efforts short of achieving elimination (so-called “sustained malaria control”) will both fail to build on success and risks losing ground.

There is ample evidence demonstrating that when prevention measures are not maintained, a resurgence in malaria transmission, illness, and deaths is predictable, sometimes occurring in just a few years. There have been at least 75 recorded resurgences of malaria transmission and disease since the 1930s, nearly all linked to decreases in funding and programmatic efforts.^7^ A concerted effort to scale up malaria interventions that will reduce morbidity and mortality while seeking the explicit goal of eliminating malaria transmission is the only logical approach for national malaria programs. There must be a defined, achievable end point to high-intensity, high-cost national malaria program investments, and that end point is elimination.

There is no dichotomy between control and elimination; there is no opting for one approach over the other. It is not possible for malaria control to be a programmatic endpoint or goal.^8^ Malaria control is the iterative application of strategic approaches by which national programs progressively diminish malaria transmission and subsequently achieve their goal of elimination. And while clearing infections and achieving malaria elimination will not lead to cost savings in the near term, it will safeguard the early gains against illness, severe disease, and death, and it and can have substantial health, economic, and societal benefits in the longer term.^9^

While the global community may be debating the goal of malaria programs, many endemic countries understand that gains are fragile and that there is a limited window of opportunity to fight malaria. They have committed to the goal of elimination in their national strategic plans and are already working with partners to pilot new strategies and tools to build the evidence base and discover what works. It is a groundbreaking effort. The next few years will be incredibly exciting, and now is not the time to ease back in the support of countries in this work. Any debate in the community should focus on the optimal way to reach our goal – not what the goal is.

To achieve elimination, countries are focusing on clearing malaria parasites from humans. Addressing the reservoir of asymptomatic carriers of the malaria parasite will be critical to stopping transmission. A consensus program implementation package for malaria elimination will soon begin to evolve from the experiences of several African nations, for example Zambia, Rwanda, Senegal, and Ethiopia. The SUFI approach has been employed to rapidly bring down malaria transmission intensity and reduce the need for and ultimately improve case management; transmission is next brought to lower, more focal levels, through large-scale programmatic approaches that build on and further expand vector control and the use of drugs, including mass drug administration and strategic treatment to block transmission.

While currently available tools have been demonstrated to support attaining elimination in areas of Asia and the Americas, strategic approaches for navigating beyond initial program scale-up in Africa have not yet been defined based on documented country program experiences. It is understood that the feasibility of transmission elimination must be systematically documented across the spectrum of transmission epidemiology in Africa.

The global malaria community is faced with an array of challenges: supporting countries that have yet to embark on early program scale-up, providing needed science to inform program policy and regulatory requirements for elimination, investing in specific new tools focused on transmission reduction, and building the human capacity and information network that will be required for countries to achieve their goal of elimination. But the arsenal of tools and program strategies available for controlling malaria is greater than ever before, and greater than generally advertised. Continued investments to optimize the innovative use of existing vector control, drugs, and prevention approaches and to develop enhanced interventions, new drugs, and totally new tools such as vaccines will be essential over the coming years to achieve elimination, especially in Africa.

Malaria elimination is proving to be a substantial undertaking. Mothers and their children in countless villages around the world deserve our dedication to this challenge.

## Footnotes

^‡^
SUFI has been agnostic to the specific tools deployed in the package of efficacious interventions but emphasizes high population coverage to achieve high levels of program effectiveness for burden reduction (morbidity and mortality) and transmission reduction.
